# The Evaluation of Ocular Posterior Segment Findings in 5527 Term Infants Using Smartphone-Based Fundus Imaging

**DOI:** 10.1155/joph/4065885

**Published:** 2024-12-24

**Authors:** Damla Erginturk Acar, Armagan Ozgur

**Affiliations:** Department of Ophthalmology, Ankara Bilkent City Hospital, Ankara, Turkey

**Keywords:** abnormal ocular findings, retinal hemorrhages, retinal white lesions, smartphone-based imaging system

## Abstract

**Purpose:** To evaluate the two-year fundus examination outcomes of term infants undergoing eye screening.

**Methods:** Retrospective review of our data of term infants at a tertiary care center (Ankara Bilkent City Hospital) from October 2021 to October 2023. All screened infants underwent red reflex test and dilated posterior segment examination. Abnormal ocular findings were documented using smartphone-based imaging system.

**Results:** A total of 5527 full-term babies were enrolled to the study. Abnormal ocular findings were observed in 1031 newborns (18.6%), the most common of which were retinal white lesions in the peripheral retina (13%) (*n* = 720) and posterior segment hemorrhages (4.3%) (*n* = 243). Other findings included congenital hypertrophy of the retinal pigment epithelium (*n* = 14), choroidal nevus (*n* = 11), idiopathic peripheral retinal scar (*n* = 9), chorioretinal coloboma (*n* = 6), optic nerve coloboma (*n* = 4), familial exudative vitreoretinopathy (*n* = 4), optic nerve large cup (*n* = 2), optic nerve hypoplasia (*n* = 2), retinal calcification (*n* = 2), optic nerve pit (*n* = 2), morning glory disc anomaly (*n* = 1), vascular loop on the optic disc (*n* = 1), retinoblastoma (*n* = 1), X-linked retinoschisis (*n* = 1), congenital toxoplasmosis (*n* = 1), thread-shaped white lesion (*n* = 1), combined hamartoma of the retina and the retinal pigment epithelium (*n* = 1), foveal hypoplasia (*n* = 1), retinal dystrophy (*n* = 1), and astrocytic hamartoma (*n* = 1).

**Conclusions:** Detailed eye examinations of term infants can reveal a range of ocular and/or systemic abnormalities that would not be caught through pupillary red reflex test. Smartphone-based fundus imaging is a simple and effective method for documenting findings.

## 1. Introduction

Newborn eye screening involves examining the eyes to detect ocular abnormalities that may require referral to an ophthalmologist. The sine qua non of screening is to carry out red reflex test at birth or thereafter as stated by the World Health Organization [1].

Ocular pathologies such as cataract, glaucoma, and retinoblastoma can be identified earlier through these screenings. In most cases, visual potential and school performance can be optimized via surgical or nonsurgical means [2, 3].

Our national eye screening program recommends that all term infants should have an ophthalmic examination carried out by their family physician or pediatrician, including pupillary red reflex test as well as assessment of strabismus by 0–3 months of age. Prompt referral to an ophthalmologist should be considered if a pathology is suspected. However, studies have showed that the red reflex examination may fail to detect a significant proportion of posterior segment diseases [4]. Detection of diseases in the first months is paramount importance in that the prognosis for some conditions, such as familial exudative vitreoretinopathy (FEVR) or retinoblastoma, depends on early intervention [5].

Recently, the use of digital fundus photography for retinopathy of prematurity (ROP) screening has become popular, even for full-term screening, providing support for consultations in challenging cases and also addressing medico legal concerns [6]. Taking a retinal photograph of a baby is not easy without special devices like RetCam and Optos, which are both expensive and unavailable in most hospitals [7]. In comparison to conventional fundus imaging systems, smartphone-based fundus imaging is cost-effective, accessible, and easy to use approach. Besides, it has been shown that retinal irradiance from modern smartphones is lower than that from an indirect ophthalmoscope, making it a safe tool for eye examination [8].

The aim of our study was to describe the posterior segment findings in term infants examined using do-it-yourself smartphone-based fundus camera in a tertiary care center.

### 1.1. Materials and Methods

The study was designed and conducted in accordance with the Declaration of Helsinki with the approval of the ethics committee. The written informed consent was obtained from each parent(s) before examination. The study was a retrospective observational study reviewing all non-premature infants who underwent neonatal ophthalmological examination at Ankara Bilkent City Hospital, between October 2021 and October 2023. Our hospital provides tertiary and quaternary neonatal intensive care services and is the largest diagnosis treatment and certified training center for ROP in Turkey.

The inclusion criteria were (1) full-term newborns birth weight ≥ 2000 g; (2) post-menstrual age between 36 and 42 weeks; and (3) APGAR score ≥ 9. Infants with a history of intensive care unit, infants with anterior segment pathologies, and infants whose parents refused to participate in the study were excluded. Complete medical information about the gestational period and delivery, gestational age at birth, birth weight, and time of examination was recorded. All newborns conforming to the criteria underwent a detailed eye examination including red reflex test and dilated posterior segment examination. Newborns were examined within first three months of life by experienced ophthalmologists (DEA or AO), with a nurse present.

Pupillary light reflex examinations were carried out using a direct ophthalmoscope. Then, pupil was dilated using 2.5% phenylephrine (Mydfrin, Alcon, USA) and 0.5% tropicamide (Tropamid, Bilim Pharmaceuticals, Turkey) eye drops for 3 times one hour before the fundus examination. Topical anesthetic (Alcaine, 0.5% proparacaine hydrochloride, Alcon, USA) was instilled to conjunctival sac a few minutes prior to sterile pediatric eyelid speculum placing. A smartphone and/or do-it-yourself smartphone-based fundus imaging device with condensing lenses (iPhone 11, Apple, USA, and Volk^®^ Digital Clearfield ClearField and Ocular Maxfield^®^ 20D condensing lenses) were used by the examiners (DEA and AO) for documentation of posterior segment findings. The smartphone's video mode was used to record clear image.

### 1.2. Statistical Analysis

For all analyses, the IBM-SPSS Version 25.0 was used. Data were presented as frequency and percentage or mean ± SD.

## 2. Results

Between October 2021 and October 2023, 16,684 term infants were born at our hospital, and 5041 of these, which is 30.2%, underwent eye screening in our clinic. Together with 486 newborns from other hospitals, a total of 5527 term infants, 2869 female (51.91%) and 2658 male (48.09%), underwent eye screening during the study period. 48 parents refused examination.

Mean gestational age (GA) was 39.40 ± 0.83 (38–41) weeks and mean birth weight (BW) was 3475.70 ± 281.72 (2640–4250) g. Out of the total number of infants, 4720 (85.4%) underwent examination between the ages of one week and two months, while 807 (14.6%) underwent examination between the ages of two and three months. Following the examinations, 19 newborns experienced a mild fever and 11 newborns exhibited conjunctival hemorrhage. However, these complications were not long-lasting.

**Figure 1:**
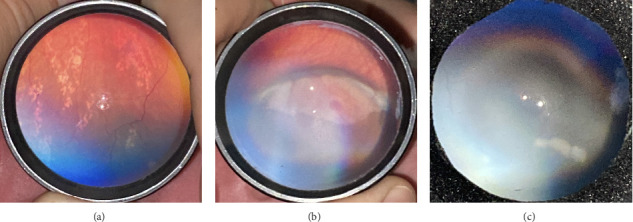
Hypopigmented retinal white lesions. (a) Hypopigmented retinal white lesion with spot-shaped. (b) Hypopigmented retinal white lesion with strip-shaped. (c) Hypopigmented retinal white lesion with patch-shaped.

**Figure 2:**
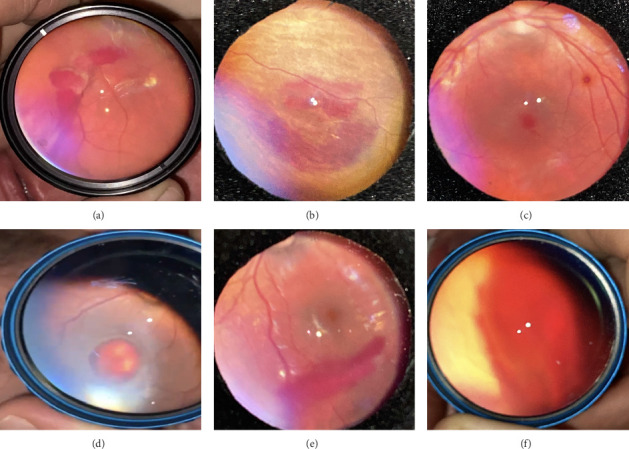
Fundus hemorrhages. (a) Optic disc hemorrhage. (b, c) Retinal hemorrhage. (d) Subhyaloid hemorrhage. (e, f) Vitreous hemorrhage.

**Figure 3:**
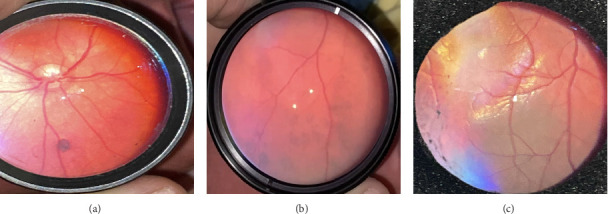
Other ocular abnormalities: (a, b) congenital hypertrophy of the retinal pigment epithelium and (c) choroidal nevus.

**Figure 4:**
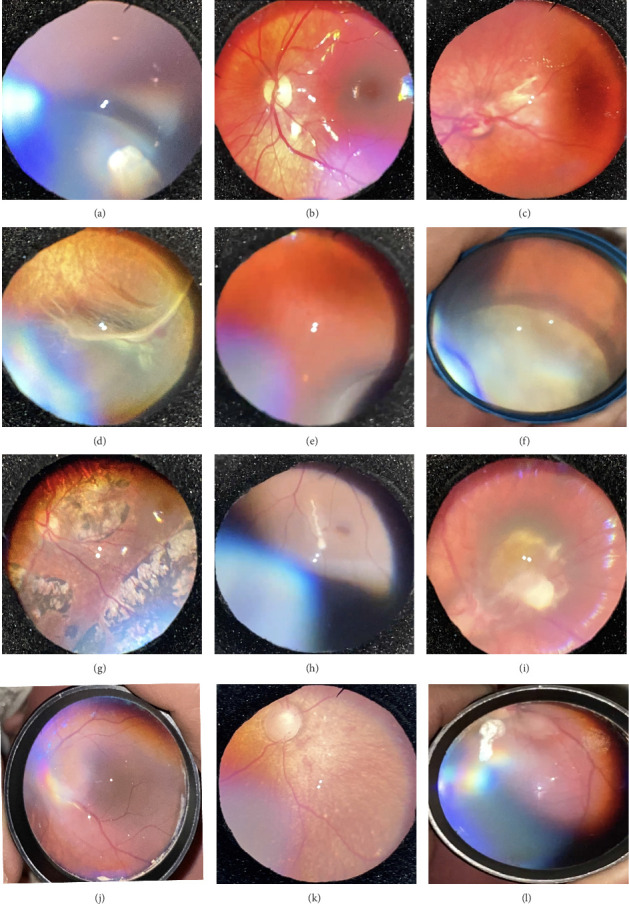
Retinal abnormalities: (a) idiopathic peripheric retinal scar, (b, c) chorioretinal coloboma, (d) FEVR, (e) retinal calsification, (f) X'linked retinoschisis, (g) congenital toxoplasmosis, (h) thread-shaped white lesion, (i) combined hamartoma of the retina and the retinal pigment epithelium, (j) foveal hypoplasia, (k) retinal dystrophy, and (l) astrocytic hamartoma.

**Figure 5:**
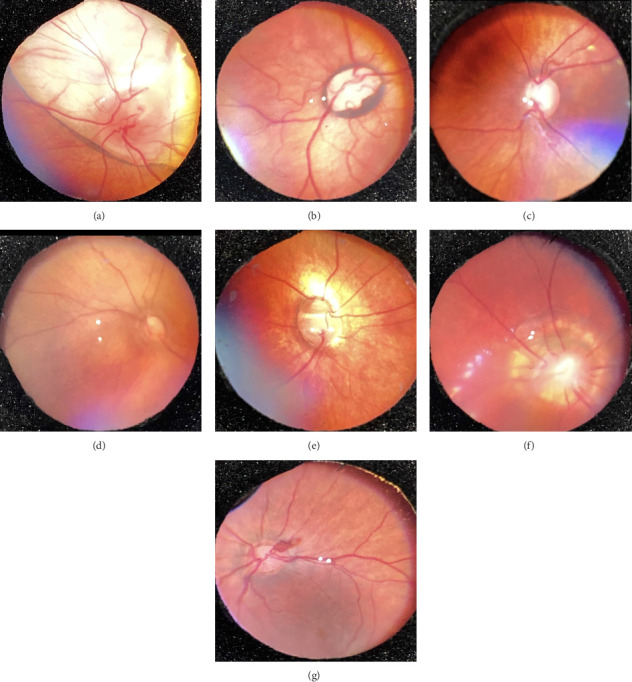
Optic disc abnormalities: (a, b) optic nerve coloboma, (c) optic nerve large cup, (d) optic nerve hypoplasia, (e) optic nerve pit, (f) morning glory disc anomaly, and (g) vascular loop on the optic disc.

**Figure 6:**
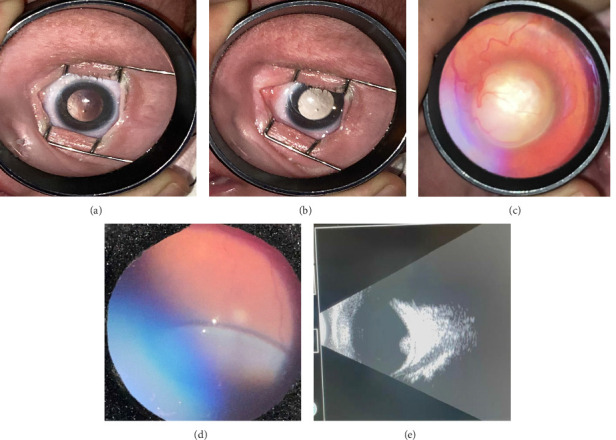
(a–e) Retinoblastoma.

Of all the infants examined, 1031 (18.7%) showed an abnormality in at least one eye. Hypopigmented retinal white lesions (Figures 1(a), 1(b), and 1(c)) were the major finding, present in 722 (13.1%) infants; they were of varying size and shape, such as spots (Figure 1(a)), stripes (Figure 1(b)), or patches (Figure 1(c)). 243 infants (4.4%) were diagnosed with fundus hemorrhage, making it the second most frequent ocular finding(Figures 2(a), 2(b), 2(c), 2(d), 2(e), and 2(f)). The hemorrhages were located in the optic disc (Figure 2(a)), retina (Figures 2(b) and 2(c)), subhyaloid area (Figure 2(d)), and vitreous (Figures 2(e) and 2(f)). Of the 243 cases, the mid-peripheral retina was affected in 153 (63%) cases, followed by the peripheral retina in 68 (28%) cases, and the entire retina in 22 cases (9%). Bilateral involvement was observed in 69.1% of cases (*n* = 168). Foveal region was involved in four babies (Figures 2(c) and 2(d)). Other findings included congenital hypertrophy of the retinal pigment epithelium (CHRPE) (*n* = 14) (Figures 3(a) and 3(b)), choroidal nevus (Figure 3(c)) (*n* = 11), idiopathic peripheric retinal scar (Figure 4(a)) (*n* = 9), chorioretinal coloboma (Figures 4(b) and 4(c)) (*n* = 6), optic nerve coloboma (Figures 5(a) and 5(b)) (*n* = 4), FEVR (*n* = 4) (Figure 4(d)), retinal calcification (Figure 4(e)) (*n* = 2), optic nerve large cup (Figure 5(c)) (*n* = 2), optic nerve hypoplasia (Figure 5(d)) (*n* = 2), optic nerve pit (Figure 5(e)) (*n* = 2), morning glory disc anomaly (Figure 5(f)) (*n* = 1), vascular loop on the optic disc (Figure 5(g)) (*n* = 1), retinoblastoma (Figures 6(a), 6(b), 6(c), 6(d), and 6(e)) (*n* = 1), X-linked retinoschisis (Figure 4(f)) (*n* = 1), congenital toxoplasmosis (Figure 4(g)) (*n* = 1), thread-shaped white lesion (Figure 4(h)) (*n* = 1), combined hamartoma of the retina and the retinal pigment epithelium (Figure 4(i)) (*n* = 1), foveal hypoplasia (Figure 4(j)) (*n* = 1), retinal dystrophy (Figure 4(k)) (*n* = 1), and astrocytic hamartoma (Figure 4(l)) (*n* = 1). These findings are summarized in Table 1. Red reflex abnormality was found in only a small minority of infants (12 of 1031 cases) (Table 2). Two patients have been diagnosed FEVR, and one patient has been diagnosed with CHARGE syndrome based on genetic evaluation. Two patients with hemorrhage were diagnosed as bleeding diathesis, one patient with inactive retinitis was diagnosed as toxoplasmosis, and one patient with optic nerve hypoplasia was diagnosed as endocrine abnormalities.

## 3. Discussion

An eye screening program of healthy term newborns is not common in most developing countries and even in some developed countries [2]. In our country, there is a national eye screening program that has been implemented since 2016. This program is based on red reflex testing performed by a neonatologist or pediatrician shortly after birth and formal visual function assessment around three years of age thereafter. Performing of red reflex test to detect ocular pathologies is relatively straightforward; however, this test may be insufficient in detecting small lesions such as foveal hemorrhage, retinoblastoma, or FEVR [4, 9].

The high cost and lack of indirect use when capturing fundus photos may restrict the use of the RetCam system's in neonatal eye screenings. We have designed a do-it-yourself, costless, and handheld, smartphone-based fundus imaging device to capture photos of fundus pathologies, thereby causing less stress and minimizing infection risks. We have also observed that thorough documentation plays a key role in enhancing communication with parents, resulting in better adherence to follow-up.

Ocular findings may range from innocuous signs to serous signs which may threaten vision and/or life [10]. In our study, the most frequent findings were retinal white lesions, which were considered innocuous finding accounting for 13% of all screened newborns. These lesions were found 17% of all screened babies in a study [11]. The classic lesion phenotype was characterized by discrete small patches of varying sizes at the level of the retinal pigment epithelium and inner retinal layers near to the ora serrata. Spots and stripe shapes were also observed. Unless there is a suspicion of retinoblastoma, ROP, or FEVR, further examination and follow-up are generally not required. Fundus fluorescein angiography and handheld optic coherence tomography would be helpful in differential diagnosing; however, these devices did not exist in our clinic. We observed that the majority of retinal white lesions resolved without sequela before two years of age so that long-term follow-up is controversial. Although the exact reason behind this pathology remains unclear, the most suggested mechanism is developmental delay in retinal vascular epithelial cells in the peripheral retina, resulting in retinal exudations due to immature development of the blood–retinal barrier [11].

The second most common pathology in our study was posterior segment hemorrhages, accounting for 4.4% of all screened newborns. The prevalence of neonatal posterior segment hemorrhages can vary widely in the studies (2%–50%); the earlier the examination, the higher the prevalence of posterior segment hemorrhages [12]. The mean postnatal examination time in our study was relatively late when compared to other studies. Studies indicated that vision impairment is more likely occur with prolonged, foveal hemorrhages; however, in our study, all hemorrhages had resolved spontaneously within 4–12 weeks, and no permanent damage was observed [13, 14]. Severe hemorrhages warranted a systemic workup. No significant hematologic abnormality was observed. A theory was put forth by Yanli et al. to explain the mechanism behind retinal hemorrhages in newborns after a spontaneous vaginal delivery. The sudden increase in intracranial pressure during vaginal delivery is caused by the compression of the fetal head as it descends. Increased pressure in the central retinal vein and dilation of the scalp and intracranial veins occur simultaneously due to obstruction of venous return. In cases where the retinal vascular structures are thin, this pressure increase may cause retinal hemorrhages [15].

The results of our study and the studies in the literature on this subject are summarized in Table 3 [5, 11, 16–21].

Ocular pathologies such as retinoblastoma are time sensitive, delayed intervention of which may lead to irreversible damage to vision either because of amblyopia or anatomic defect and may threaten life [22]. FEVR is an inherited vitreoretinopathy characterized by congenital abnormal retinal vascularization. Retinal exudates, neovascularization, retinal folds, preretinal membranes, and retinal detachment may result in visual loss [23, 24]. We found an avascular area as well as peripheral fibrovascular proliferation in one eye of a baby born at 38 weeks and 3150 g, and prompt laser was carried out. We ruled out incontinentia pigmenti and Norrie's disease because of the absence of hearing problems and skin symptoms and requested genetic consultation. The family members of these cases underwent retinal evaluation, and no retinal pathology was detected.

The early management of retinoblastoma may improve the visual and survival outcome. In general, retinoblastoma can be diagnosed above 1 year of age in the absence of a family history [25]. We diagnosed a patient with no family history in the first month of life. The leukocoria was so hardly visible that (Figure 6(a)) we thought that this case could not have been noticed by red reflex test alone.

Neuroimaging, endocrine investigations, or detailed genetic tests are recommended for pathologies such as congenital anomalies of the optic disc [26, 27]. We diagnosed different congenital anomalies in our study, resulting in early intervention and reasonable outcomes.

Our study has a limitation. It is not a multicenter study; therefore, we cannot generalize our findings to the entire population. Our study has indicated that a considerable portion of infants exhibit ocular anomalies, pointing toward the possibility of hundreds of thousands of infants being affected on a national level.

In conclusion, detailed eye examinations of term infants can reveal a range of ocular and/or systemic abnormalities that would not be caught through pupillary red reflex test. Smartphone-based fundus imaging is a simple and effective method for documenting findings.

## Figures and Tables

**Table 1:** Findings of fundus abnormalities of 1031 cases in term newborns.

Fundus abnormalities found on newborn screening	Number of cases	Percentage out of 5527 infants
Hypopigmented retinal white lesion	722	%13.1
Retinal hemorrhage	243	%4.4
Congenital hypertrophy of the retinal pigment epithelium (CHRPE)	14	%0.002
Choroidal nevus	11	%0.001
Idiopathic peripheric retinal scar	9	%0.001
Chorioretinal coloboma	6	%.0.001
Optic nerve coloboma	4	%0.0007
Familial exudative vitreoretinopathy	4	%0.0007
Optic nerve large cup	2	%0.0003
Optic nerve hypoplasia	2	%0.0003
Retinal calcification	2	%0.0003
Optic nerve pit	2	%0.0003
Morning glory disc anomaly	1	%0.0001
Vascular loop on the optic disc	1	%0.0001
Retinoblastoma	1	%0.0001
X-linked retinoschisis	1	%0.0001
Congenital toxoplasmosis	1	%0.0001
Thread-shaped white lesion	1	%0.0001
Combined hamartoma of the retina and the retinal pigment epithelium	1	%0.0001
Foveal hypoplasia	1	%0.0001
Retinal dystrophy	1	%0.0001
Astrocytic hamartoma	1	%0.0001

**Table 2:** Red reflex test abnormalities.

Abnormalities	Number of cases	Percentage out of 5527 infants
Retinal hemorrhage	3	%0.0005
Chorioretinal coloboma	2	%0.0003
Optic nerve coloboma	2	%0.0003
Morning glory	1	%0.0001
Retinoblastoma	1	%0.0001
Combined hamartoma	1	%0.0001
Toxoplasmosis	1	%0.0001
X-linked retinoschisis	1	%0.0001

**Table 3:** The studies in the literature evaluating the fundus examination findings of newborns.

Study	Country	Population	Abnormality prevalence (%)	The two most common reasons
Tang et al. [16]	China	199,851	9.11	Retinal hemorrhage Abnormal fundus pigmentation
Liu, Zheng, and Lu [17]	China	23,861	27.8	Retinal hemorrhage Retinal exudate
Li et al. [18]	China	15,284	21	Retinal hemorrhage Subretinal lipid or calcium deposition
Yenice, Petriçli, and Kara [19]	Turkey	2972	6.2	Retinal hemorrhage Retinal white change
Sitorus et al. [20]	Indonesia	1208	12.4	Retinal hemorrhage Chorioretinitis
Vinekar et al. [5]	India	1021	4.7	Retinal hemorrhage A “ridge” resembling retinopathy of prematurity
Luo et al. [21]	China	779	12.8	Retinal hemorrhage Retinal exudate
Ma et al. [11]	China	481	41.2	Retinal white change Retinal hemorrhage
Present study	Turkey	5527	18.6	Retinal white change Retinal hemorrhage

## Data Availability

The data that support the findings of this study are available on request from the corresponding author. The data are not publicly available due to privacy or ethical restrictions.
